# Burden of falls in China, 1992–2021 and projections to 2030: a systematic analysis for the global burden of disease study 2021

**DOI:** 10.3389/fpubh.2025.1538406

**Published:** 2025-03-21

**Authors:** Liang Sui, Yueming Lv, Kai Xin Feng, Fu Jie Jing

**Affiliations:** School of Acupuncture-Tuina, Shandong University of Traditional Chinese Medicine, Jinan, Shandong, China

**Keywords:** falls, epidemiology, global burden of disease study, gender disparities, China

## Abstract

**Background:**

The escalating burden of falls in China necessitates a detailed examination to elucidate its dynamics and trends. Using data from the Global Burden of Disease Study (GBD) 2021, this research assessed the burden of falls in China.

**Methods:**

Data from GBD 2021 were analyzed using Joinpoint regression to identify long-term trends. The impact of mortality and disability-adjusted life years (DALYs) rate for falls was investigated through the age-period-cohort model. Additionally, a decomposition analysis was performed to ascertain the distinct impacts of population growth, aging, and epidemiological changes on the burden of falls from 1992 to 2021. Furthermore, this study employed both the BAPC and Nordpred models to project future burdens of falls.

**Results:**

From 1992 to 2021 in China, the age-standardized rates of falls showed divergent trends. Prevalence and incidence rates increased, while mortality rates generally decreased. Males consistently exhibited higher rates than females. The rates of prevalence, incidence, and mortality exhibit a sharp increase beyond the age of 75 in 2021. Decomposition analysis identified aging as the primary driver of increased prevalence and mortality, particularly in females. Joinpoint regression analysis revealed fluctuating trends in prevalence and incidence with periods of increase and decline, and a general decrease in mortality except during brief intervals. DALYs and years of life lost (YLLs) rates generally decreased, with intervals of stabilization and minor increases, while years lived with disability (YLDs) showed significant fluctuations. By 2030, the projected DALYs rate for falls is expected to rise to approximately 547.4 per 100,000. Fractures of the lower extremity predominated as the leading cause of disability post-fall, with hip fractures increasingly contributing to disability among the older adult. Additionally, from 1992 to 2021, the population attributable fraction (PAF) of low bone mineral density for DALYs due to falls increased to 23.2%, with the PAF reaching 33.3% among women in 2021.

**Conclusion:**

Falls continue to significantly burden public health in China. Our findings highlight the urgent need to develop targeted prevention and intervention strategies that cater to the country’s unique demographic characteristics, aiming to mitigate the growing public health impact of falls.

## 1 Introduction

The World Health Organization characterizes falls as incidents that unintentionally bring a person to the ground, floor, or a lower level, and they rank as the second leading cause of accidental death worldwide (1, 2). Falls rank among the most frequent injury mechanisms and consistently present a threat to health and longevity across various age groups. In younger, otherwise healthy individuals, falls can result in lifelong disabilities, including spinal cord damage or traumatic brain injuries, and often result in critical injuries that necessitate sophisticated surgical treatments, such as complex bone fractures or repair of intra-abdominal organ injuries (3). In older adults, the adverse effects of falls are often intensified by concurrent conditions such as reduced bone density, osteoporosis, or the administration of antiplatelet or anticoagulant therapies (4, 5). Falls represent a significant global public health challenge, heavily impacting health and medical care costs (6). Annually, an estimated 37.3 million severe falls occur worldwide, resulting in approximately 17 million disabling injuries and 684,000 fatalities due to falls, with over 80% of these incidents taking place in low- to middle-income countries (1).

Falls pose a significant threat to the health and well-being of people in China. In 2019, around 9.76 million severe falls were recorded among individuals aged 60 and older, leading to a cumulative loss exceeding 3.18 million disability-adjusted life years (DALYs) (7). This number is expected to rise as the population continues to age. National statistics indicate that around 40 million older adult individuals in China experience at least one fall annually, with direct medical costs exceeding 5 billion yuan each year (8). Despite the growing burden, research on falls among the population in China remains limited, although existing studies highlight the substantial health and economic impact of falls (1, 7, 9).

This study aims to comprehensively analyze the burden and trends of falls in China from 1992 to 2021. Specifically, we seek to examine the changes in the burden of falls over the past three decades. Analyze the 2021 data to identify which age and gender groups are most vulnerable. Apply joinpoint regression to assess trends and perform a decomposition analysis to explore contributing factors. Investigate the nature of injuries caused by falls to understand the specific health impacts. Conduct age-period-cohort analyses to understand temporal and generational shifts in fall-related mortality and DALYs rates. Project future trends up to 2030. Investigate associated risk factors to identify potential areas for intervention. By addressing these objectives, this study will provide critical insights into the epidemiology of falls in China, supporting targeted prevention and intervention efforts to mitigate this growing public health concern.

## 2 Methods

### 2.1 Data source

The study utilized data from the Global Burden of Disease (GBD) 2021 study, providing comprehensive health metrics categorized by sex, age, and region, updated annually through international cooperation. The report details 88 risk factors, 371 diseases, and 288 causes of death and injuries across 204 nations (10). Extensive methodological details of the GBD 2021 study have been described in prior research (10, 11). In China, primary data were derived from censuses, Disease Surveillance Points, population surveys, and the Chinese CDC’s Cause of Death Reporting System, and systematic reviews assessing disease incidence and prevalence (12). Data regarding the incidence, prevalence, mortality, and DALYs, including years lived with disability (YLDs) and years of life lost (YLLs), and their age-standardized rates (ASRs) for falls were sourced from the Global Health Data Exchange (https://vizhub.healthdata.org/gbd-results/). Concept Definition and Retrieval Strategy related to disease burden in this study can be found in the Supplementary material.

### 2.2 Join-point

Long-term trends in the burden of falls, were analyzed by assessing average annual percent changes (AAPC) with 95% confidence intervals (CIs) using a joinpoint regression model (version 5.2.0; National Cancer Institute, USA). This model divides the time series into distinct segments and detects significant trends within each segment (13, 14). The AAPC is calculated by weighting the regression coefficients from each segment’s annual percent changes. A positive AAPC, with a 95% CI that does not include zero, suggests an increasing age-standardized rate (ASR). Conversely, a negative AAPC, with a 95% CI that remains below zero, indicates a declining ASR.

### 2.3 Decomposition analysis

To clarify the key factors driving changes in the burden of falls from 1992 to 2021, a decomposition analysis was utilized. This method aimed to assess the distinct impacts of epidemiological shifts, aging and population growth (15, 16). The analysis involved estimating the impact of each factor while holding the other two constant.

### 2.4 Age-period-cohort modeling analysis

In this analysis, the age-period-cohort (APC) model was employed to investigate the impact of age, time period, and birth cohort on the burden of falls in China. For this purpose, data on mortality and DALYs from falls were organized across continuous five-year intervals from 1992 to 2021. The APC model utilized a log-linear approach to model the rates, incorporating additive effects from birth cohorts, calendar periods, and age. In the model, age effects illustrate variance in risk across age groups; period effects capture uniform temporal shifts affecting all groups; and cohort effects reflect risk variations among peers born during the same timeframe (17–19). We designated the median birth cohort, period group, and age group as reference categories, choosing the lower median when categories were even in number (20). The R package from the Biostatistics Branch of the NIH, USA, was used for modeling and deriving estimable functions (20).

### 2.5 BAPC model projection

This study utilized the Bayesian age-period-cohort (BAPC) model, an extension of the conventional generalized linear model that integrates Bayesian principles for dynamic age, period, and cohort analysis (21). We performed the BAPC model using the BAPC package (version 0.0.36) in R (version 4.4.2, available at http://www.r-project.org). The widespread verification and use of the BAPC model in epidemiological studies, particularly those examining age-structured populations with complex cohort dynamics underscore its efficacy (22).

### 2.6 Nordpred model projection

The Nordpred model, which builds on the APC framework, accurately projects future burdens for diseases or injuries by analyzing trends and demographic shifts, including population structure changes and generational impacts (23). For this analysis, we used the Nordpred APC model, implemented through the nordpred package (version 1.1), to estimate DALYs associated with falls from 2022 to 2030.

## 3 Results

### 3.1 Trends in the burden of falls in China from 1992 to 2021

In 2021, China experienced a substantial burden of falls, with a total prevalence of 111,705,688 cases, marking a 104.24% increase since 1992. The prevalence was more pronounced in men, accounting for 62,270,730 cases, compared to 49,434,958 cases in women. The incidence of falls stood at 39,776,772 cases for the year, a 59.25% rise from 1992, with women reporting 17,546,171 cases versus 22,230,601 cases in men. Regarding deaths, there were 141,657 fatalities due to falls, an 88.07% increase from 1992. Men accounted for 85,053 deaths and women for 56,604. DALYs for falls totaled 8,301,289, an increase of 39.7% over the past three decades, split between 5,106,057 for men and 3,195,232 for women. YLDs due to falls were reported at 4,821,165 person-years, up by 89.63% since 1992, with women contributing 2,202,854 person-years and men 2,618,310 person-years. YLLs from falls reached 3,480,124, showing a modest increase of 2.36%, where men had 2,487,747 and women accounted for 992,377 person-years.

The prevalence rate of falls per 100,000 population was 7,851.41, an increase of 73.16% since 1992. Men had a higher prevalence rate of 8552.46 per 100,000, while women registered a rate of 7116.6 per 100,000. The incidence rate stood at 2795.77 per 100,000 population, up by 35.02% from 1992, with women showing a rate of 2525.93 per 100,000 compared to men’s 3053.22 per 100,000. The mortality rate was 9.96 per 100,000 in 2021, a 59.45% rise since 1992, with men displaying a higher rate of 11.68 per 100,000 than women’s 8.15 per 100,000. The DALYs rate stood at 583.47 per 100,000, up by 18.44% from 1992, with rates for women and men at 459.98 and 701.28 per 100,000, respectively. The YLDs rate was 338.86 per 100,000 in 2021, a 60.77% rise since 1992, with men displaying a higher rate of 359.61 per 100,000 than women’s 317.12 per 100,000. The YLLs rate was 244.61 per 100,000, decreasing by 13.21% over the years, with women and men showing rates of 142.86 and 341.67 per 100,000, respectively. Between 1992 and 2021, falls in China showed a declining trend in the ASRs for DALYs, YLLs and deaths. In contrast, the ASRs for prevalence, incidence, and YLDs exhibited an increasing trend during the same period. The gender-specific data summarized in Table 1 reveal a higher disease burden in males compared to females.

**Table 1:** Description analysis of burden of falls from 1992 to 2021.

Measure	Sex	All-ages number in thousands, 2021, n (95% UI)	All-ages cases changes, 1992–2021, (%)	All-ages rates per 100,000 people, 2021 (95% UI)	All-ages rates changes, 1992–2021 (%)	Age-standardized rates per 100,000 people, 2021 (95% UI)	Age-standardized rates changes, 1992–2021 (%)
Incidence	Male	22230.60 (20003.94, 24750.45)	48.08	3053.22 (2747.4, 3399.3)	26.75	3072.97 (2746.55, 3449.05)	28.69
	Female	17546.17 (15756.64, 19615.75)	76.08	2525.93 (2268.31, 2823.86)	47.78	2361.39 (2097.99, 2683.72)	32.99
	Both	39776.77 (35757.32, 44260.73)	59.25	2795.77 (2513.26, 3110.94)	35.02	2748.04 (2453.03, 3101.97)	30.52
Prevalence	Male	62270.73 (53807.96, 71043.15)	91.91	8552.46 (7390.15, 9757.29)	64.27	6873.49 (5967.58, 7846.62)	19.36
	Female	49434.96 (43200.79, 56518.55)	122.23	7116.6 (6219.14, 8136.35)	86.52	5282.18 (4612.16, 6036.15)	23.11
	Both	111705.69 (97251.67, 127663.20)	104.24	7851.41 (6835.49, 8973.01)	73.16	6116.41 (5325.81, 6972.99)	20.67
Deaths	Male	85.05 (53.14, 114.96)	68.91	11.68 (7.3, 15.79)	44.58	11.52 (7.25, 15.32)	−8.73
	Female	56.60 (31.91, 77.66)	126.7	8.15 (4.59, 11.18)	90.27	6.06 (3.45, 8.3)	−16.81
	Both	141.66 (91.10, 183.32)	88.07	9.96 (6.4, 12.89)	59.45	8.64 (5.54, 11.09)	−12.74
YLLs	Male	2487.75 (1648.55, 3307.33)	2.5	341.67 (226.42, 454.24)	−12.27	319.93 (216.49, 419.19)	−26.61
	Female	992.38 (574.99, 1350.11)	2.03	142.86 (82.78, 194.36)	−14.37	118.77 (70.3, 159.44)	−42.39
	Both	3480.12 (2344.16, 4493.21)	2.36	244.61 (164.76, 315.81)	−13.21	220.29 (149.56, 281)	−31.99
YLDs	Male	2618.31 (1814.22, 3584.31)	79.09	359.61 (249.17, 492.28)	53.3	286.63 (197.21, 394.46)	9.28
	Female	2202.85 (1493.64, 3017.62)	103.88	317.12 (215.02, 434.41)	71.12	231.86 (156.9, 318.91)	8.58
	Both	4821.16 (3291.15, 6625.63)	89.63	338.86 (231.32, 465.69)	60.77	261.52 (178.01, 360.3)	8.57
DALYs	Male	5106.06 (3981.54, 6439.55)	31.29	701.28 (546.84, 884.43)	12.38	606.56 (476, 759.7)	−13.13
	Female	3195.23 (2431.12, 4129.62)	55.63	459.98 (349.98, 594.5)	30.62	350.63 (267.7, 446.33)	−16.45
	Both	8301.29 (6363.99, 10467.99)	39.7	583.47 (447.3, 735.76)	18.44	481.81 (374.1, 600.84)	−14.69

**Figure 1:**
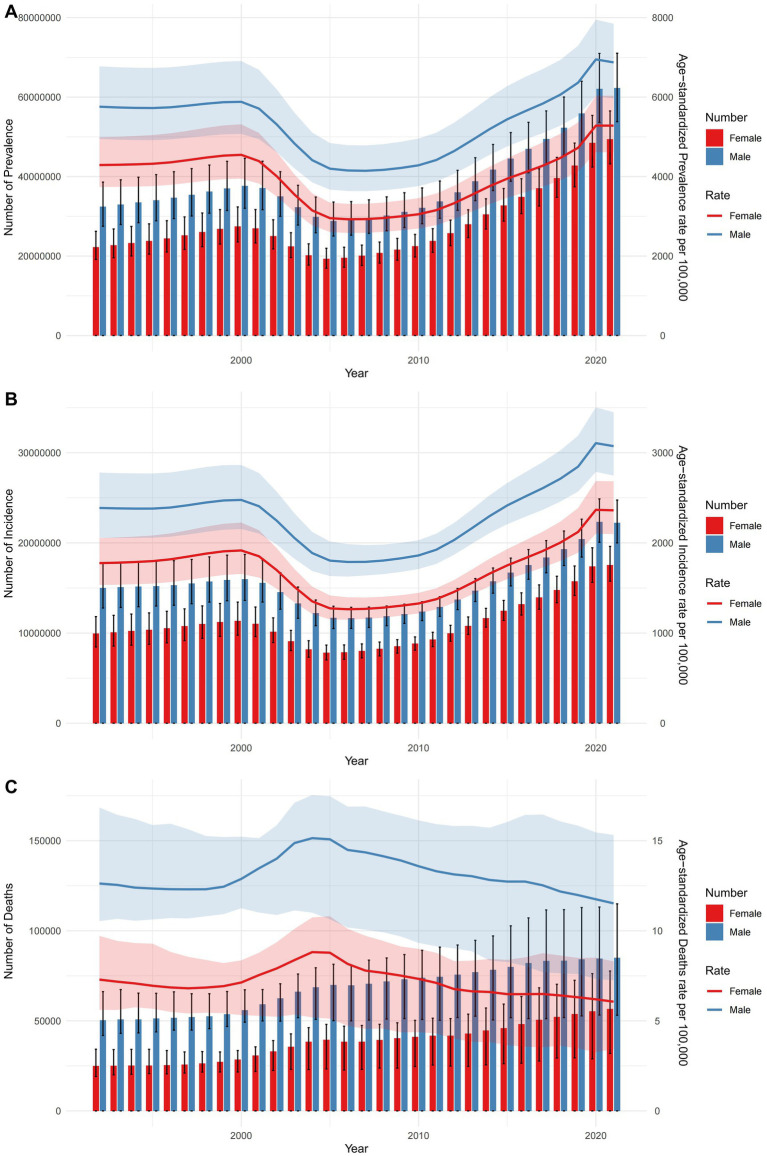
Trajectories of the age-standardized rates and all-age numbers of prevalence, incidence, and mortality due to falls by sex from 1992 to 2021. **(A)** Prevalence. **(B)** Incidence. **(C)** Deaths.

Figure 1 displays sex-specific, all-age numbers and age-standardized rates for falls in terms of prevalence, incidence, and mortality throughout China from 1992 to 2021. The data indicate an upward trend in the numbers of cases, incidences, and deaths due to falls, although these figures have fluctuated over the years. While there has been a general increase in prevalence and incidence rates across sexes, mortality rates have shown a declining trend during this period. Males consistently demonstrated higher age-standardized rates across all metrics compared to females. Further details on falls-related DALYs, YLDs, and YLLs are provided in Supplementary Figures 1A–C.

### 3.2 Disease burden of falls in China in 2021 by age and gender

**Figure 2:**
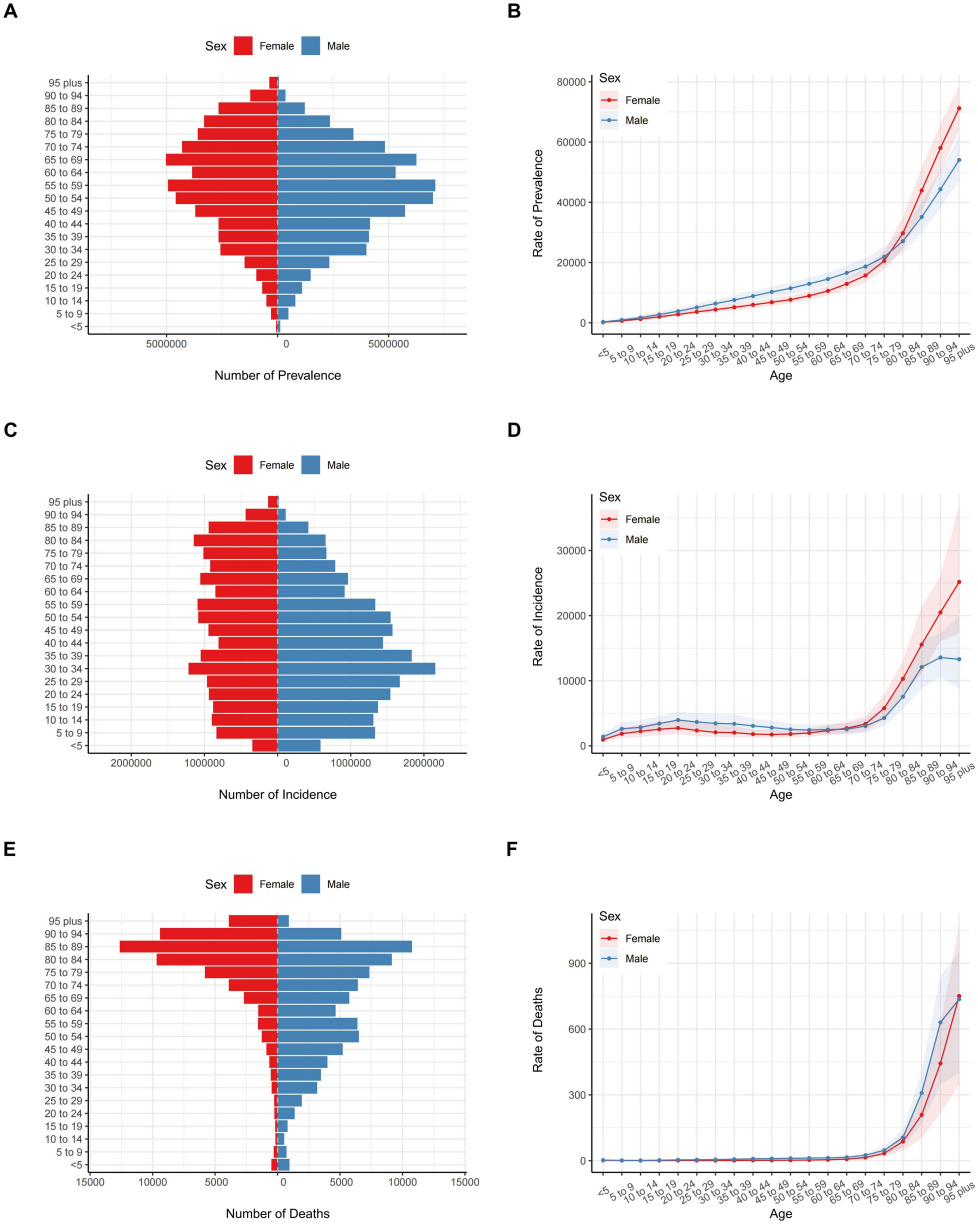
Numbers and rates of prevalence, incidence, and mortality due to falls among different age groups in China in 2021. **(A)** Number of prevalence. **(B)** Rate of prevalence. **(C)** Number of incidence. **(D)** Rate of incidence. **(E)** Number of deaths. **(F)** Rate of deaths.

Figure 2 illustrates the numbers of prevalence, incidence, and mortality (panels A, C, E) along with their respective rates (panels B, D, F) for falls across various age groups in 2021. In China, the total number of falls across different age groups initially increased and then decreased, with the highest number observed in the 50 to 74 years age group for both males and females. Regarding the total number of fall incidents, the figures remained relatively consistent across age groups, with most showing fewer than one million cases, particularly for females. For males, the number of incidents initially increased and then decreased, with the highest number seen in the 25 to 39 years age group. When it comes to falls-related mortality, there was a clear increasing trend, with the highest number of fatalities observed in the 80 to 89 years age group for both males and females. Notably, the rates of prevalence, incidence, and mortality exhibit a sharp increase beyond the age of 75.

The rates and numbers of DALYs, YLDs, and YLLs, segmented by sex and age group, are detailed in Supplementary Figure 2. In terms of DALYs due to falls, the total count showed an increasing trend, peaking in the 65 to 89 years age group, particularly among females. For males, the DALYs count initially increased and then decreased, with the highest number observed in the 45 to 59 years age group. The total YLLs due to falls followed a U-shaped curve, with a small peak in the under-9 years age group and the highest number of YLLs in the 75 to 94 years age group for females. In males, the YLLs count also followed a U-shaped pattern, with a small peak in the under-9 years age group and the highest YLLs count in the 30 to 59 years age group. The YLDs due to falls showed an initial increase followed by a decrease, with the highest number of YLDs observed in the 50 to 74 years age group for both males and females. Notably, the rates of DALYs, YLDs, and YLLs exhibit a sharp increase beyond the age of 75.

### 3.3 Trajectories in disease burden of falls in China based on joinpoint regression analysis

**Figure 3:**
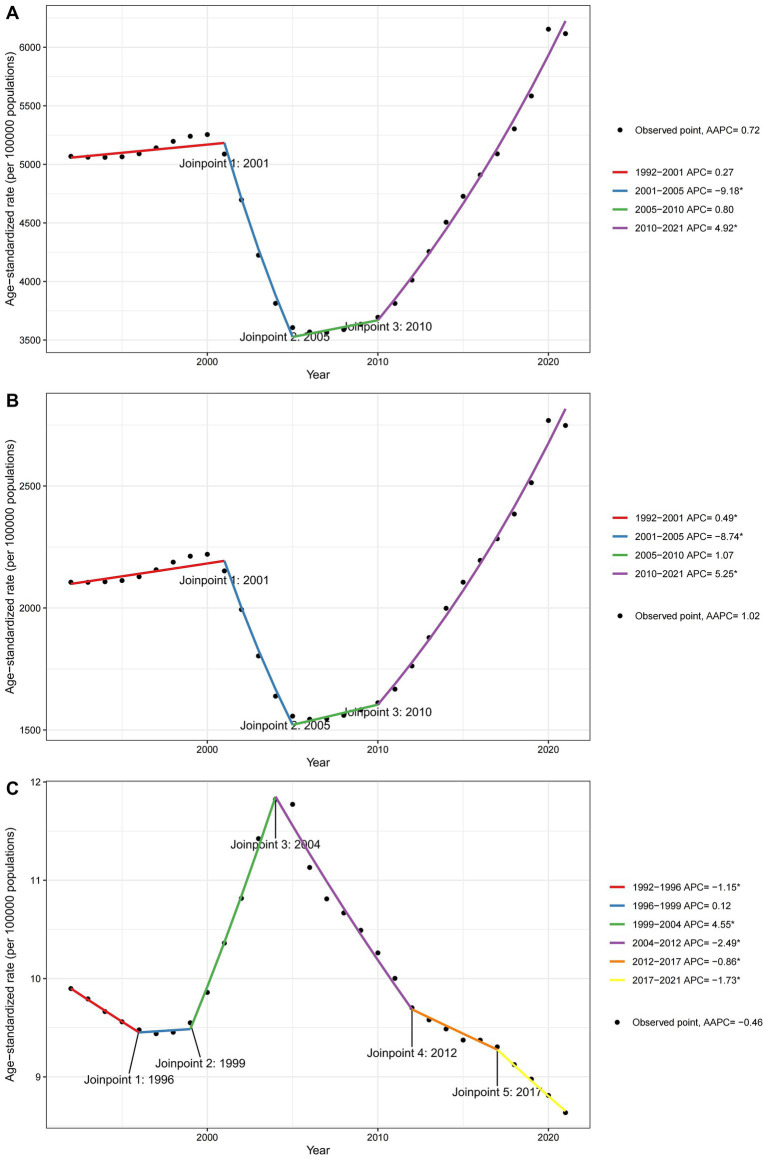
Joinpoint regression analysis of ASRs for falls in China over the period 1992 to 2021. **(A)** Prevalence, **(B)** Incidence, and **(C)** Mortality.

Figure 3 presents the joinpoint regression analyses of the ASRs for prevalence, incidence, and mortality of falls in China from 1992 to 2021. We observed that from 1992 to 2001, the prevalence exhibited a slight increasing trend (APC = +0.27). From 2001 to 2005, there was a significant decline in prevalence (APC = −9.18). The prevalence then showed a slight increase from 2005 to 2010 (APC = +0.8), followed by a significant rise from 2010 to 2021 (APC = +4.92). The trends in incidence rates were similar to those in prevalence. Regarding mortality rates, there was a decreasing trend from 1992 to 1996 (APC = −1.15), a slight increase from 1996 to 1999 (APC = +0.12), and a significant increase from 1999 to 2004 (APC = +4.55). From 2004 to 2021, the mortality rates continuously declined (APC = −2.49 from 2004 to 2012, APC = −0.86 from 2012 to 2017, and APC = −1.73 from 2017 to 2021). Supplementary Figure 3 presents joinpoint regression analyses of ASRs for incidence, prevalence, and mortality across both sexes from 1992 to 2021. The analysis reveals consistent trends in these rates across both genders over the observed period.

**Figure 4:**
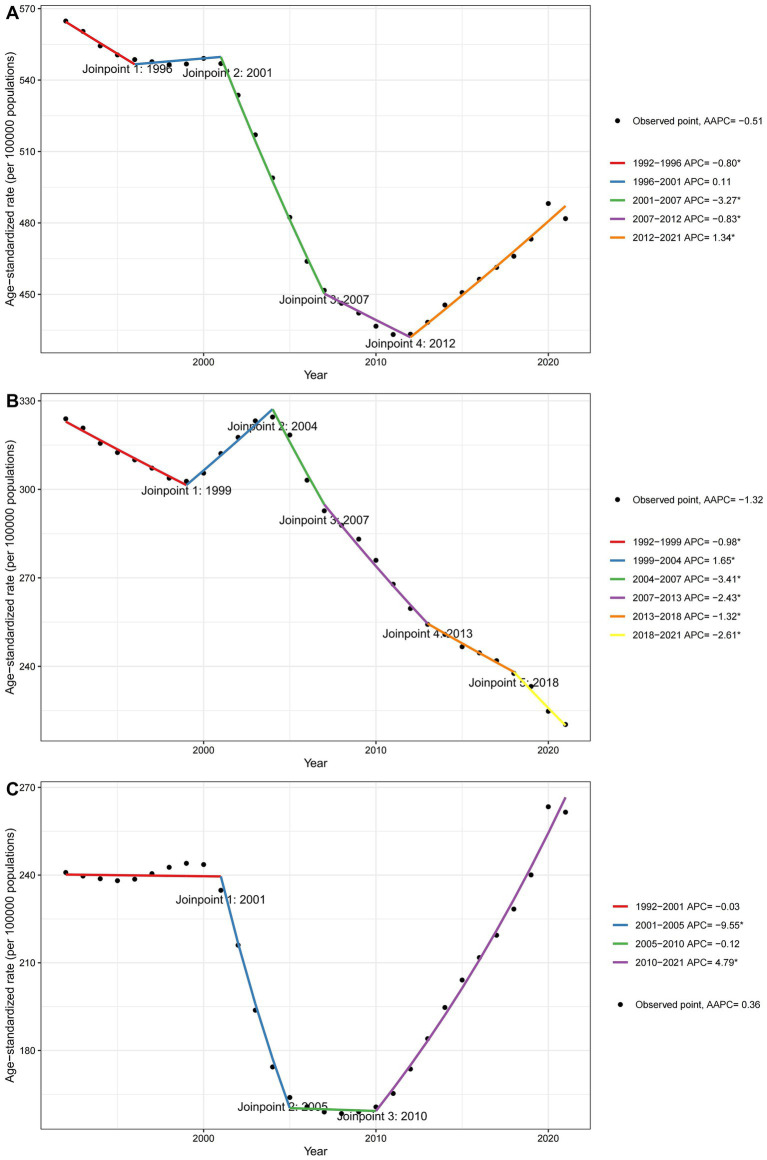
Joinpoint regression analysis of ASRs for falls in China over the period 1992 to 2021. **(A)** DALYs, **(B)** YLLs, and **(C)** YLDs.

Joinpoint regression analyses of the ASRs for DALYs, YLLs, and YLDs of falls in China from 1992 to 2021 are depicted in Figure 4. For DALYs, a steady decrease is observed from 1992 to 1996 (APC = −0.80), followed by a stable phase from 1996 to 2001 (APC = +0.11). From 2001 to 2007, DALYs declined significantly (APC = −3.27), stabilizing slightly from 2007 to 2012 (APC = −0.83), before increasing again significantly from 2012 to 2021 (APC = +1.34). In Figure 4B for YLLs, the rate initially decreased from 1992 to 1999 (APC = −0.98), followed by a period of increase from 1999 to 2004 (APC = +1.65). From 2004 to 2021, the rates consistently decreased (APC = −3.41 from 2004 to 2007, APC = −2.43 from 2007 to 2013, and APC = −1.32 from 2013 to 2018). Subsequently, from 2018 to 2021, the rates experienced a significant decline (APC = −2.61). Figure 4C shows the trends for YLDs where the rates remained relatively stable from 1992 to 2001 (APC = −0.03). A significant decrease is noted from 2001 to 2005 (APC = −9.55), followed by a slight decrease from 2005 to 2010 (APC = −0.12). From 2010 to 2021, the YLDs exhibited a continuous increase (APC = +4.79). Supplementary Figure 4 presents joinpoint regression analyses of ASRs for DALYs, YLLs, and YLDs in both sexes from 1992 to 2021. The analysis reveals consistent trends in these rates across both genders over the observed period.

### 3.4 Nature of injuries caused by falls

**Figure 5:**
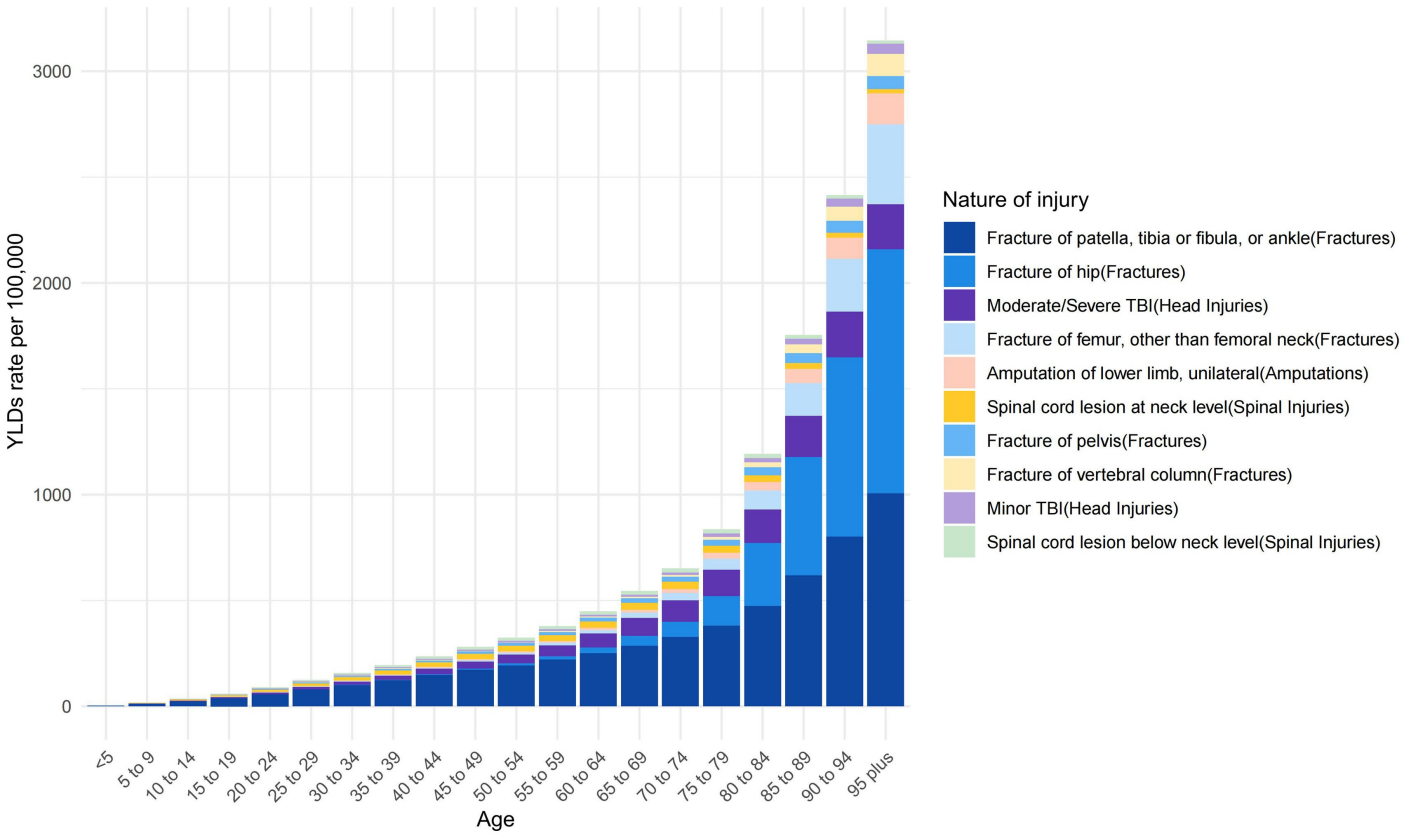
Age-specific nature-of-injury composition of falls in China.

Figure 5 presents the age-specific distribution of injury types resulting from falls in China. The figure indicates that fractures of the patella, tibia, fibula, or ankle are the predominant reasons for disability due to falls across all age groups. Nevertheless, fractures of the hip increasingly contribute to disability among the older adult.

### 3.5 Decomposition analysis

**Figure 6:**
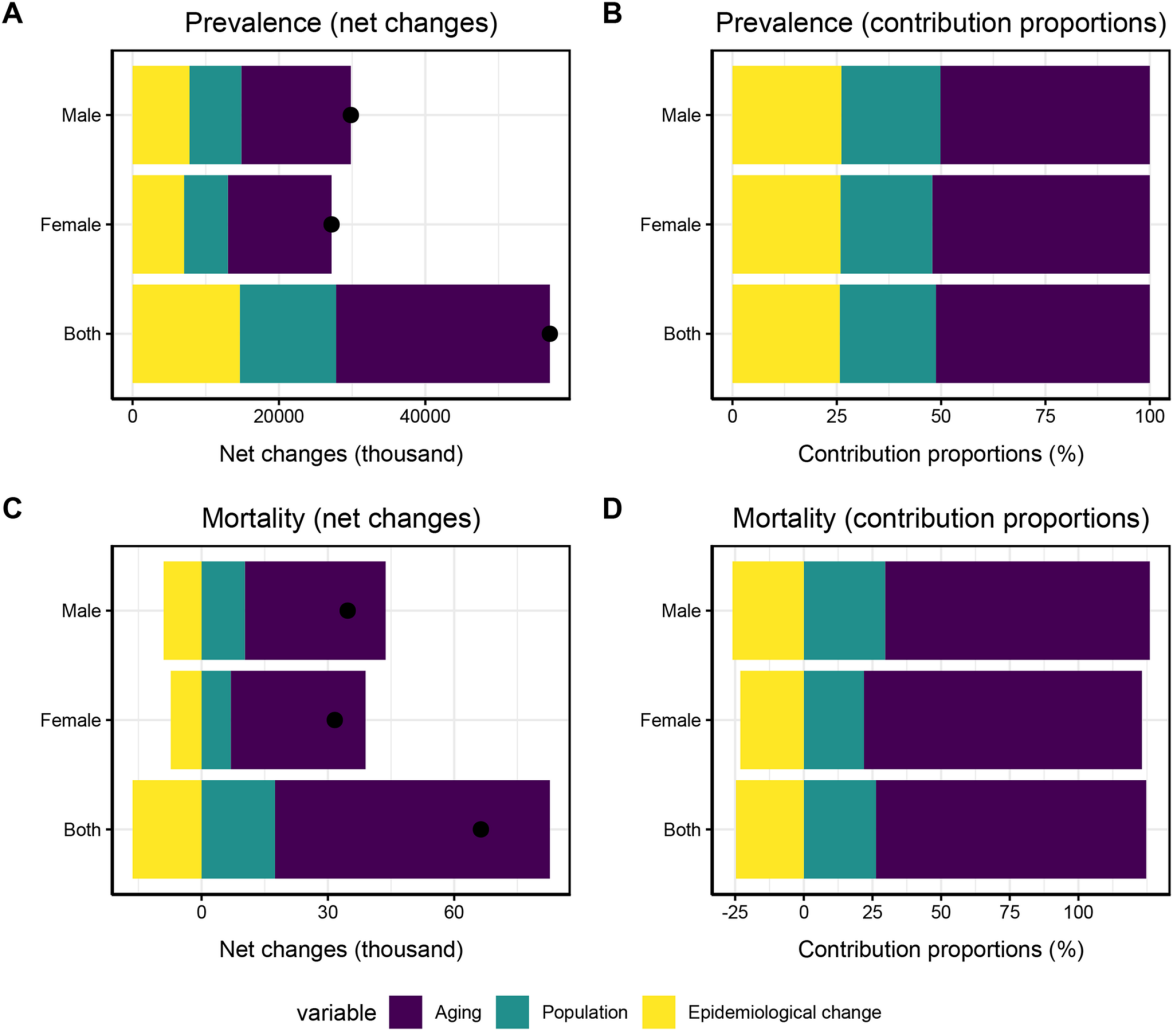
Decomposition analysis of Falls in China from 1992 to 2021. **(A)** Changes in Falls prevalence. **(B)** Proportion of the population-level determinants attributed to changes in Falls prevalence. **(C)** Changes in Falls mortality. **(D)** Proportion of the population-level determinants attributed to changes in Falls mortality.

Decomposition analysis of changes in fall prevalence over the past 30 years identified aging as the primary driver for the increase in cases, with a higher contribution observed in females (52.12%) compared to males (50.17%). Specifically, aging accounted for 52.12% (approximately 14,172,749 cases) of the total changes in prevalent cases among women and 50.17% (approximately 14,960,912 cases) among men. Epidemiological changes were the second largest contributing factor, with males (26.09%) showing a higher contribution than females (25.90%). Additionally, population growth contributed positively, accounting for 23.75% (approximately 7,082,161 cases) in men and 21.98% (approximately 5,975,713 cases) in women (Figure 6).

As per the decomposition analysis, aging was the primary positive driver for the increase in mortality numbers, contributing more significantly in females than in males (101.22% vs. 96.36%). Specifically, 101.22% (approximately 32,023.37 cases) of the total change in mortality among women and 96.36% (approximately 33,438.36 cases) among men were attributed to aging. Another important positive driver was population growth, which had a higher contribution in males compared to females (29.62% vs. 21.89%). Regarding mortality rate changes (i.e., epidemiological changes), negative contributions were observed in both sexes: −25.98% (approximately −9,014.87 cases) in males and − 23.11% (approximately −7,312.49 cases) in females (Figure 6).

### 3.6 Age–period–cohort analysis for falls mortality and DALYs rates in China

**Figure 7:**
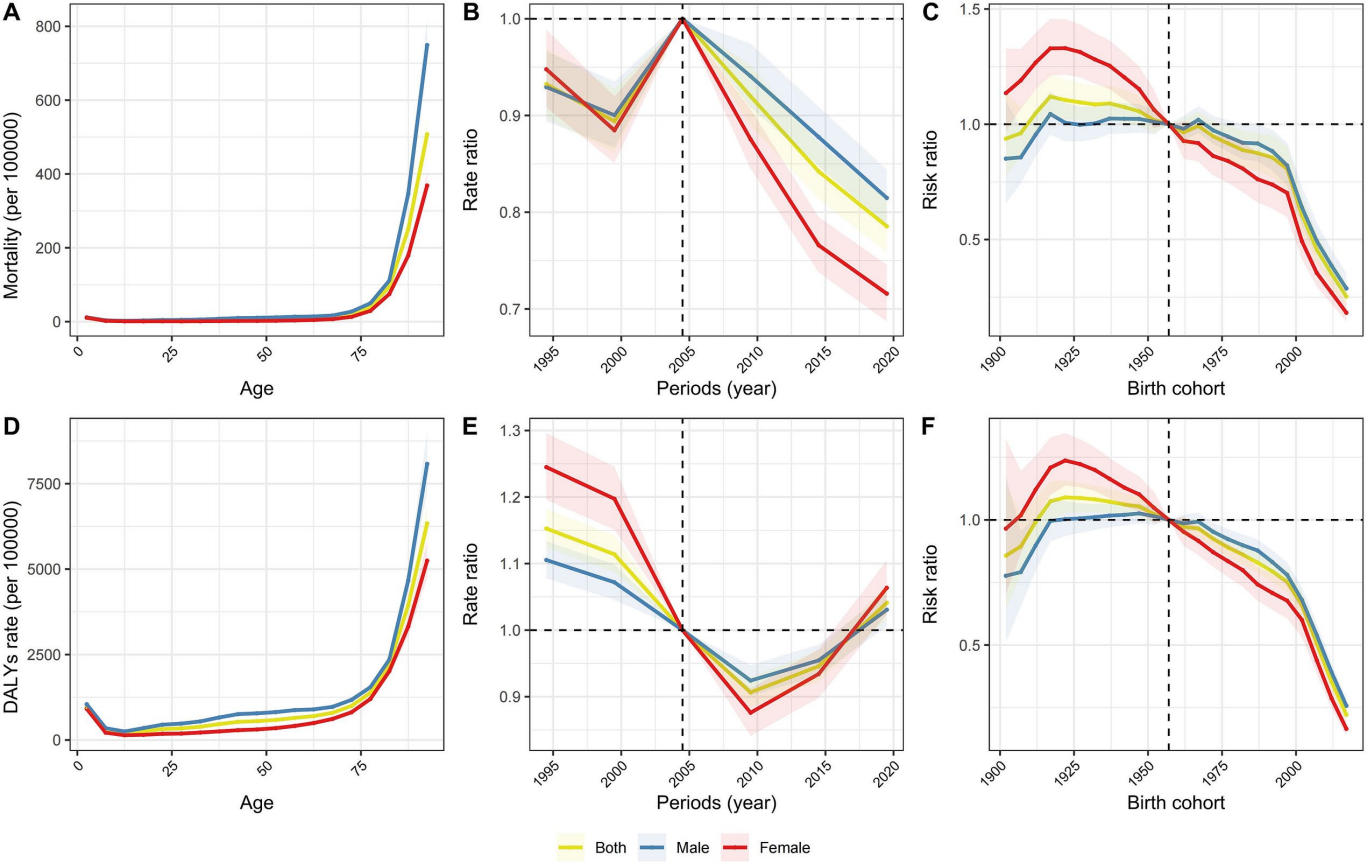
Age, period and cohort effects on falls mortality and DALY rates in China. **(A)** The age curves of falls mortality by gender. **(B)** The period RRs of falls mortality by gender. **(C)** The cohort RRs of falls mortality by gender. **(D)** The age curves of falls DALY by gender. **(E)** The period RRs of falls DALY by gender. **(F)** The cohort RRs of falls DALY by gender.

Figure 7 and Supplementary Tables present the estimated age, period, and cohort effects for falls mortality and DALYs rates. The age effects demonstrate a linear increase, peaking in later years for both mortality and DALYs rates. From 1992 to 2021, the period effects for falls mortality—overall, and specifically for both males and females—are favorable. In contrast, the DALYs rates for falls exhibit an unfavorable period effect from 1990 to 2019. Notably, however, both male and female period effects displayed favorable trends during 2007–2016. The cohort effects for mortality and DALYs rates reveal overall fluctuating downward trends throughout the period from 1992 to 2021.

### 3.7 Projections of falls DALYs rates and number for the next 9 years

**Figure 8:**
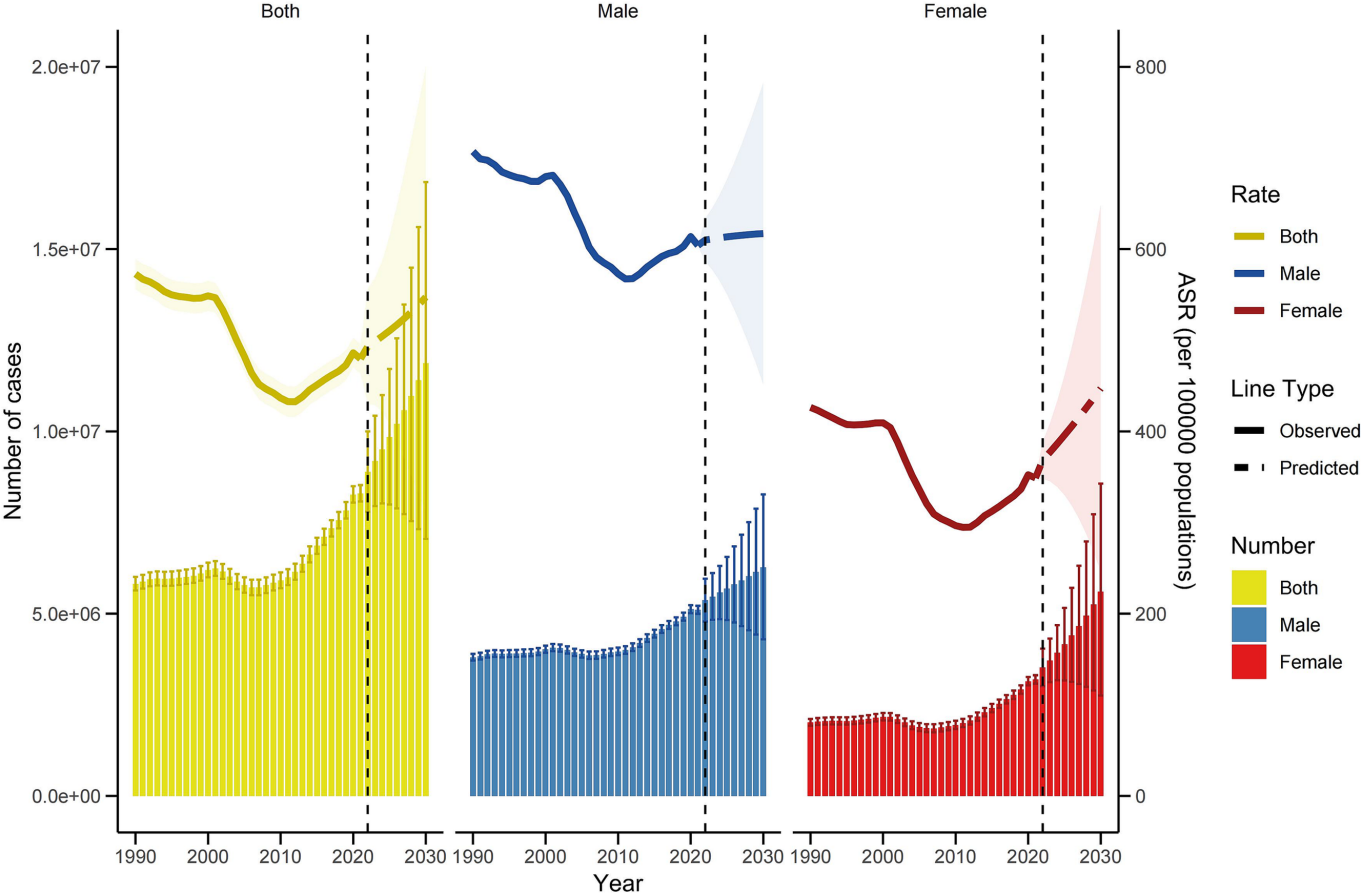
Trends in the ASRs for DALYs and numbers of DALYs by sex in China using BAPC.

Figure 8 and Supplementary Tables depict the ASRs for DALYs of falls in China from 1992 to 2030, as predicted by the BAPC model. The overall age-standardized rate declines to its lowest point in 2012, followed by a steady increase, reaching an estimated 547.4 per 100,000 people by 2030. For females, the age-standardized DALYs rate follows a ‘U-shaped’ pattern, initially decreasing before rising again, with projections indicating a rate of approximately 447.1 per 100,000 people by 2030. In males, a similar trend is observed, with the rate expected to increase to about 617.0 per 100,000 by 2030. The total number of falls-related DALYs is projected to continue rising, peaking at approximately 11,872,183 by 2030. This upward trajectory is mirrored in the gender-specific analysis, with female and male DALYs expected to peak at about 5,602,737 and 6,269,447, respectively.

**Figure 9:**
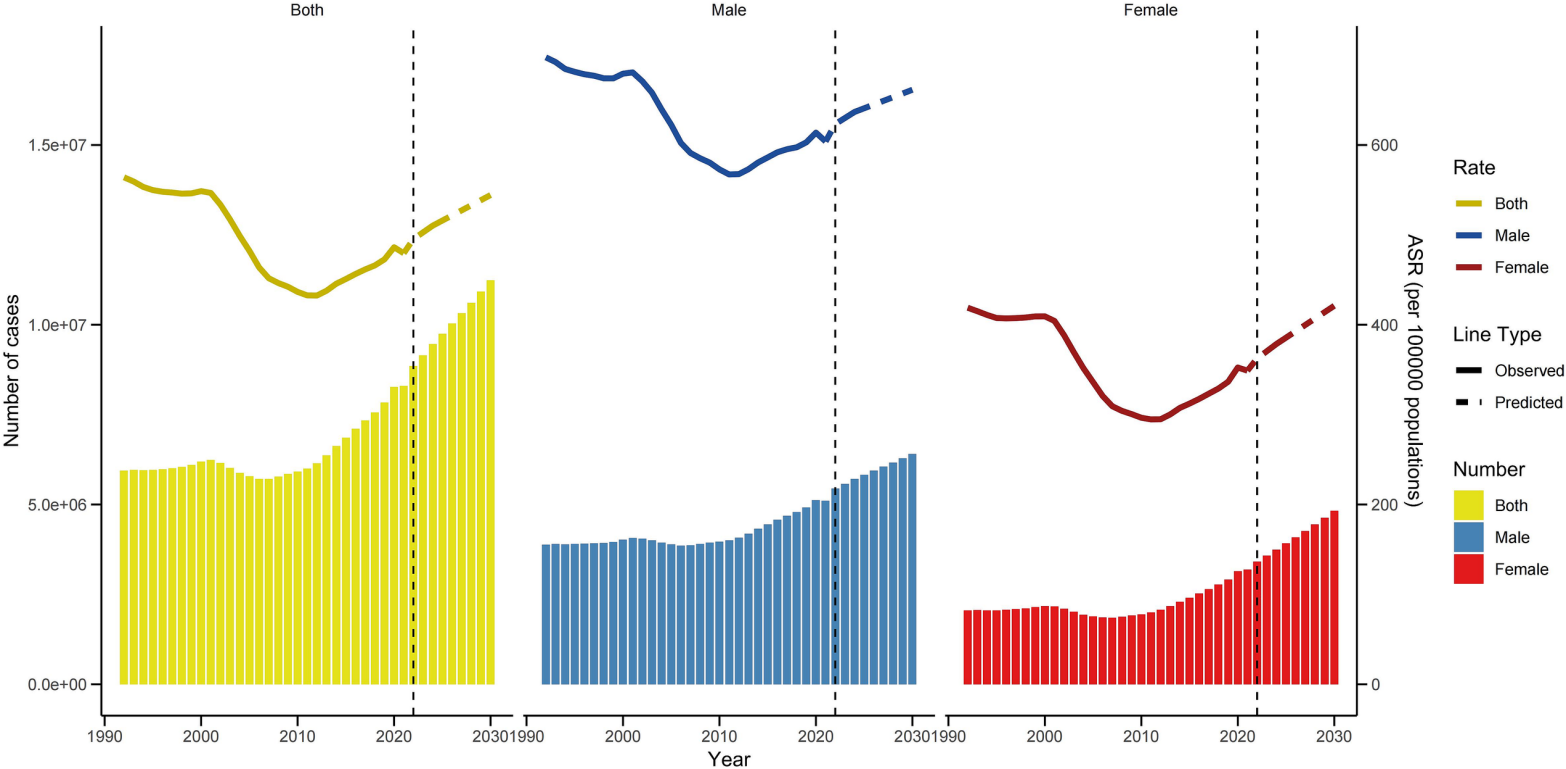
Trends in the ASRs for DALYs and numbers of DALYs by sex in China using Nordpred.

We used nordpred model to predict the future burden of falls-related DALYs in China as a sensitivity analysis. The results indicate a continuous increase in the ASRs for DALYs of falls from 2022 to 2030 (Figure 9 and Supplementary Tables). By 2030, the rate is projected to reach approximately 544.3 per 100,000 population, with the total number of falls-related DALYs rising to about 11,239,568. For females, the number of falls DALYs is expected to increase, potentially reaching 4,830,858 by 2030. Similarly, the male falls DALYs burden is also projected to rise, reaching approximately 6,408,710 by 2030.

### 3.8 Trends of associated risk factors of falls from 1992 to 2021

**Figure 10:**
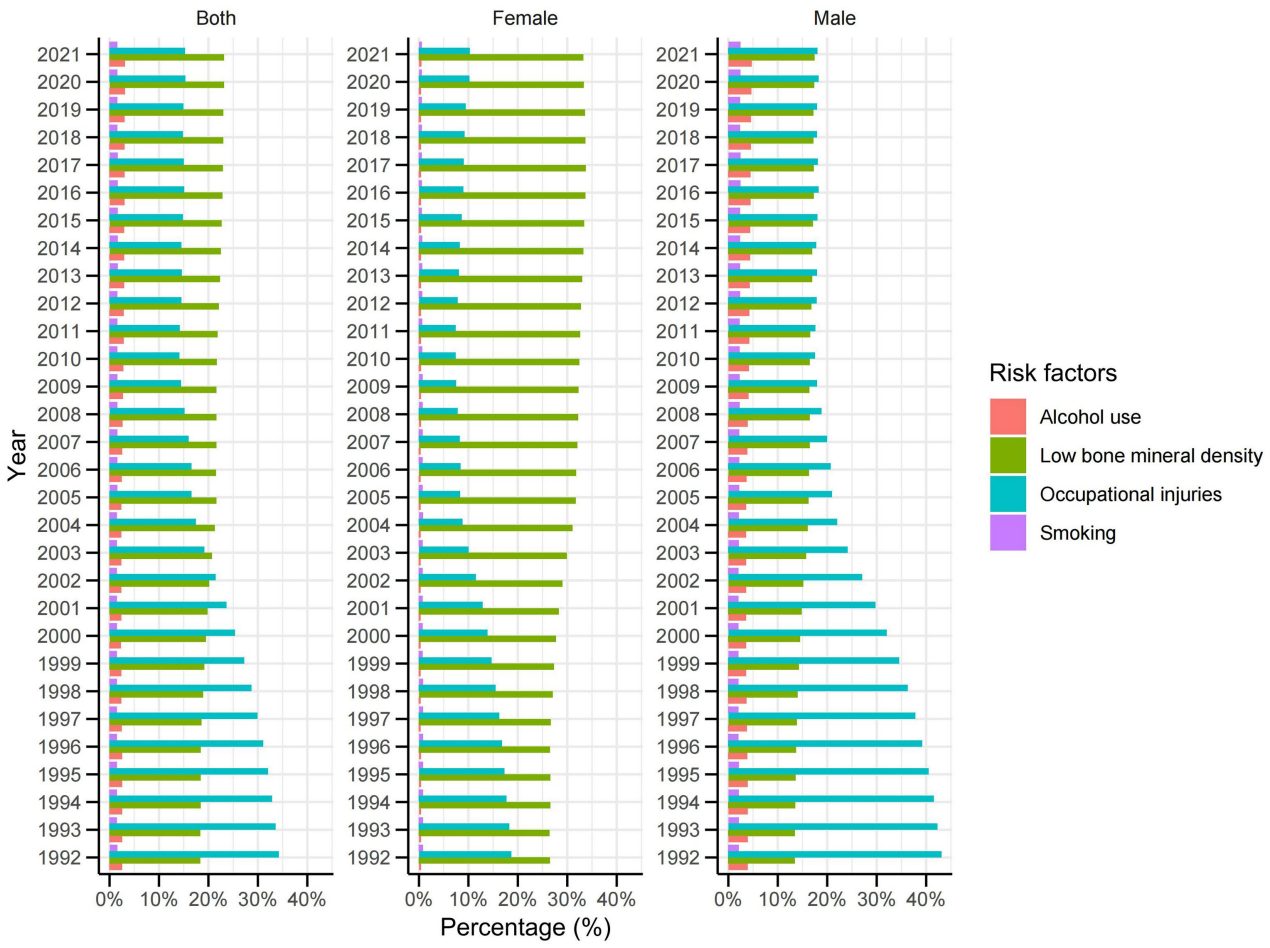
Population attributable fraction (PAF, %) of associated risk factors for falls DALYs from 1992 to 2021.

In China, the population attributable fraction (PAF) for low bone mineral density (LBMD) related to DALYs from falls increased steadily from 18.4% in 1992 to 23.2% by 2021 (Figure 10). Conversely, the PAF for occupational injuries exhibited a consistent decline over the same period, from 34.2 to 15.3%. The contributions of alcohol use and smoking to DALYs remained relatively stable and minor, each accounting for less than 5 % throughout the study period. Specifically, in 2021, the PAF for LBMD contributing to DALYs from falls was 33.3% among women, while for occupational injuries it was 18.0% among men.

### 3.9 Comparative analysis of the burden of falls globally, in the United States of America, India, and China from 1992 to 2021

**Figure 11:**
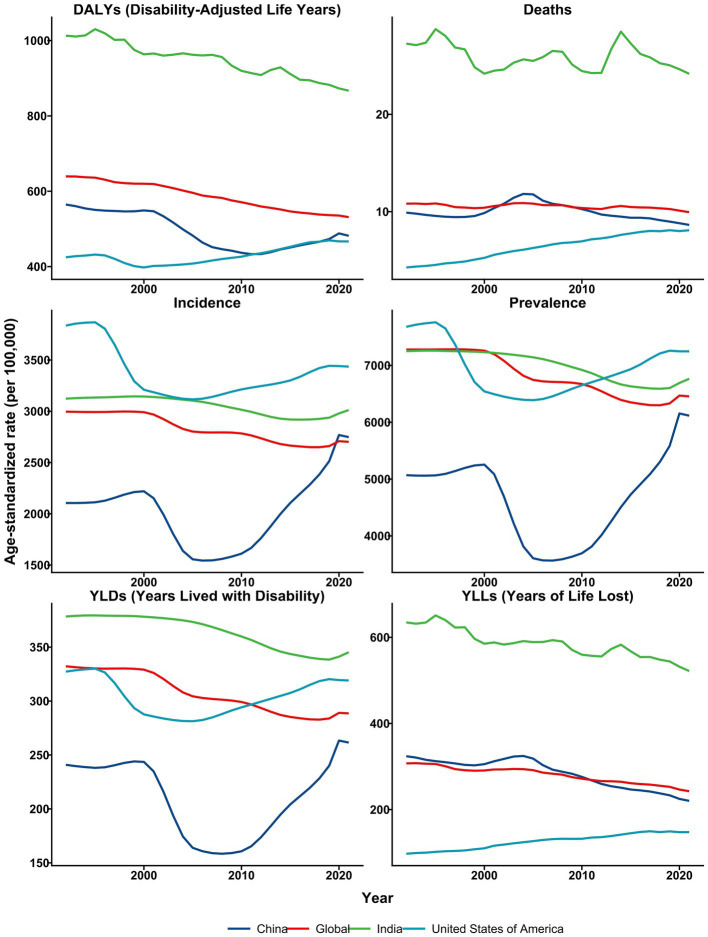
The trends in age-standardized rates of falls globally, as well as in the United States, India, and China, from 1992 to 2021.

Figure 11 shows the ASRs of fall burden globally, as well as in the United States of America, India, and China, from 1992 to 2021. During the study period, India consistently exhibited the highest DALYs rates due to falls, remaining above 900 per 100,000 in most years. The global DALYs rates gradually decreased from approximately 640 per 100,000 to 531 per 100,000. China’s DALYs burden remained slightly below the global average, while the United States had the lowest DALYs burden, starting at around 425 per 100,000 in the early 1990s and increasing to approximately 467 per 100,000 by 2021. India also had the highest mortality rate from falls, ranging from 24 to 29 deaths per 100,000, whereas the global mortality rate fluctuated between 10 and 11 deaths per 100,000. China’s mortality rate remained at or slightly below the global average in recent years, and the United States had the lowest mortality rate, which increased from 4 deaths per 100,000 in 1992 to 8 deaths per 100,000 by 2021.

Regarding incidence, the United States initially had the highest rates, exceeding 3,836 cases per 100,000 in 1992. This rate declined to 3,116 cases per 100,000 by 2005, then rose to 3,436 cases per 100,000 by 2021. India’s incidence rate slightly decreased over time, from 3,123 cases per 100,000 to 3,012 cases per 100,000. In contrast, China consistently had the lowest incidence rate, dipping below 1,544 cases per 100,000 in 2006, then increasing to 2,748 cases per 100,000 by 2021.

The United States had the highest prevalence, starting at 7,680 cases per 100,000 in 1992, decreasing to 6,389 cases per 100,000 by 2005, and then rising to 7,248 cases per 100,000 by 2021. India and the global prevalence curves followed similar trends, both gradually decreasing from approximately 7,265 cases per 100,000 in 1992. Notably, China’s prevalence initially declined but then increased, reaching its lowest point in 2007 (3,566 cases per 100,000) and rising to 6,116 cases per 100,000 by 2021.

In terms of YLDs, India had the highest value in 1992 (378 per 100,000), which decreased to 345 per 100,000 by 2021. The global YLDs rates gradually decreased from 332 per 100,000 in 1992 to 289 per 100,000 by 2021. The United States saw an initial decline in YLDs, reaching a minimum of 281 per 100,000 in 2005, before increasing to 319 per 100,000 by 2021. China’s YLDs began at 241 per 100,000, decreased to 158 per 100,000 around 2008, and then gradually increased to 262 per 100,000 by 2021.

Regarding YLLs, India had the highest values, starting at 635 per 100,000 in 1992 and decreasing to 522 per 100,000 by 2021. China’s YLLs started at 324 per 100,000 and declined to 220 per 100,000, while the global average decreased from 307 per 100,000 to 243 per 100,000. The United States had the lowest YLLs, starting at 97 per 100,000 and rising to over 148 per 100,000 by 2021.

## 4 Discussion

This longitudinal analysis of data from the GBD Study over the last three decades reveals key trends in the incidence, prevalence, mortality, and disability burden of falls in China. The study reveals significant increases in both new and existing fall rates, although mortality rates have declined. Additionally, disability burden rates exhibited gender-specific patterns, with males consistently experiencing higher rates than females. Aging is recognized as a major factor in the increasing prevalence and mortality from falls. Notably, the PAF for low bone mineral density, a major risk factor, has steadily increased from 1992 to 2021. The projected age-standardized DALYs rates of falls are expected to rise to approximately 547.4 per 100,000 individuals by 2030. This discussion seeks to interpret these findings, assess their implications for policy and practice, and recommend avenues for future research.

The increasing burden of falls among the Chinese population may be attributed to multiple factors, including an aging population (24–26), the prevalence of chronic diseases (27, 28), inappropriate environments (29), lifestyles, and a decline in physical function (30). The rise in the incidence of age-standardized rates of falls, coupled with a decline in the mortality of age-standardized rates of falls, may reflect increased awareness and reporting of falls, as well as advancements in China’s primary healthcare system and emergency medical services during the study period (31, 32).

This study’s findings align with prior research revealing the ASRs for prevalence, incidence, and mortality rates of falls have consistently been higher in males than in females over the past 30 years (33). The higher prevalence of falls in men can be linked to several risk factors. Men tend to engage in more outdoor activities, which increases their exposure to fall risks (34). Additionally, they often experience a more rapid decline in muscle strength and balance as they age, which can lead to more severe injuries when falls occur (35).

This study demonstrates that the incidence, prevalence, and mortality rates of falls increase markedly after the age of 75. Decomposition analysis reveals that aging is a primary factor contributing to the rise in fall prevalence and mortality over the past 30 years. The increased mortality from falls among individuals over 75 years of age can be attributed to several interrelated factors. One of the primary reasons is the higher prevalence of frailty and associated comorbidities in this age group. Frailty significantly increases the risk of adverse outcomes following a fall, including mortality. Studies have shown that frailty is the strongest predictor of mortality in older adults after a fall, with frail individuals experiencing notably higher mortality rates compared to their more robust peers (36). Additionally, the physiological changes associated with aging, such as decreased bone density, impaired balance, and reduced muscle strength, contribute to the severity of injuries sustained during falls. Moreover, the use of certain medications, particularly those that affect balance and cognition, can exacerbate the risk of falls and subsequent injuries. The interaction of multiple medications, known as polypharmacy, is common in older adults and can lead to side effects such as dizziness and confusion, increasing the likelihood of falls (37, 38).

The observed sex disparities in fall-related outcomes require comprehensive investigation through an age-strandified lens. The elevated incidence of falls among males in early adulthood (25–39 years) may reflect higher levels of physical activity combined with working at heights (39, 40). This pattern intensifies in middle age, as evidenced by the earlier DALYs peak in males (45–59 years) compared to females (65–89 years), potentially mirroring the cumulative exposure to occupational hazards in manual labor sectors versus the predominant influence of biological aging processes in postmenopausal females (41). The U-shaped YLLs distribution reveals critical vulnerabilities at life extremes - pediatric falls (<9 years) frequently involve high-energy mechanisms like falls from heights (42), while the secondary mortality peak in working-age males (30–59 years) suggests modifiable risk amplifiers including substance use patterns and safety protocol non-compliance in industrial settings (43, 44). The highest YLLs in females aged 75–94 years correlate with age-related physiological declines, including osteoporosis, sarcopenia, and impaired balance (45).

In our analysis, fractures of the patella, tibia or fibula, and ankle emerged as predominant causes of disability following injurious falls across all age groups. However, our study further delineates the age-related variance in fracture impact, highlighting an increased contribution to disability from hip and femur fractures in older populations. This age-related shift in fracture sites is likely attributable to changes in bone density and balance stability with advancing age (46, 47).

This study demonstrates that low bone mineral density showed a PAF that increased steadily from 1992 to 2021, in China. In 2021, the proportion of LBMD among women was 33.3%. LBMD presents a significant risk for early onset and severity in women (48), who have inherently smaller and thinner bone structures compared to men. Post-menopausal women are especially susceptible to calcium loss and osteoporosis due to decreased estrogen levels, which lack protective effects on the skeletal system. Consequently, fall prevention approaches for older adult individuals with LBMD should be gender-inclusive, considering the distinct needs and risks of both sexes.

The overall age-standardized DALYs rates of falls among the Chinese population is projected to escalate to an estimated 547.4 per 100,000 individuals by 2030. This significant increase underscores the need for comprehensive and tailored prevention and management strategies. Collaborative initiatives involving policymakers, healthcare providers, and community organizations are crucial. Effective strategies should encompass multifactorial risk assessments, home safety evaluations, medication reviews, and vision and hearing checks (49). To prevent falls in children under the age of nine, implementing safety measures such as the use of safety gates, furniture corner covers, and window locks can significantly reduce the risk (50, 51). It is essential to educate parents about potential fall hazards and effective prevention strategies, which include supervising children and modifying the home environment accordingly (52, 53). Encouraging behaviors like not leaving children unattended on high surfaces and minimizing the use of baby walkers are also important steps in fall prevention (50, 53). For the older adult, the model based on gait analysis can identify, prevent, and manage individuals at risk of falling (54). In addition, exercise programs that improve balance, strength, and mobility have been shown to effectively decrease the incidence of falls among community-dwelling older adult individuals (55–57).Gender-specific interventions are critical, with male-focused programs emphasizing risk reduction and behavioral safety, while female-focused initiatives should prioritize bone health and osteoporosis prevention, which is particularly relevant given its prevalence among older women (58, 59). Additionally, addressing environmental hazards—such as improving lighting and flooring in both private and public spaces—can significantly decrease fall risks (60, 61).

This study builds on previous research by conducting a decomposition analysis to distinguish the independent effects of aging, population growth, and epidemiological shifts on the burden of falls between 1992 and 2021. It utilized both the BAPC and Nordpred models to forecast future burdens, offering a methodological enhancement over prior studies. However, this research has several limitations that merit attention. It solely analyzes the GBD 2021 datasets within the context of China, neglecting potential variances across provinces and between urban and rural settings, which could affect the applicability of the findings across different regions. Additionally, as data collection ceased in 2021, the study may not capture recent trends that could influence the current and future burden of falls. The estimates were also derived using system dynamics and statistical modeling based on limited raw data, which might lead to potential distortions from significant assumptions required by these models. For future research, it would be advisable to extend the analysis to include more current data and explore regional differences within China. Additionally, addressing both inter-provincial differences and urban–rural disparities would yield a more holistic insight into the burden of falls across diverse demographic and geographical landscapes.

## 5 Conclusion

The burden of falls in China, both currently and in future projections, is substantial. Addressing this issue requires crucial enhancements in investment toward further research, the development of effective fall prevention initiatives, and improved healthcare accessibility.

## Data Availability

Publicly available datasets were analyzed in this study. This data can be found here: https://vizhub.healthdata.org/gbd-results/.
