# Supplementary material for: Burden of falls in China, 1992–2021 and projections to 2030: a systematic analysis for the global burden of disease study 2021
Source: Front Public Health. 2025 Mar 21;13:1538406. doi: 10.3389/fpubh.2025.1538406 (PMC11968356; doi:10.3389/fpubh.2025.1538406)
Supplement: Supplementary file 1 [file Image_1.pdf]

Supplementary Figure

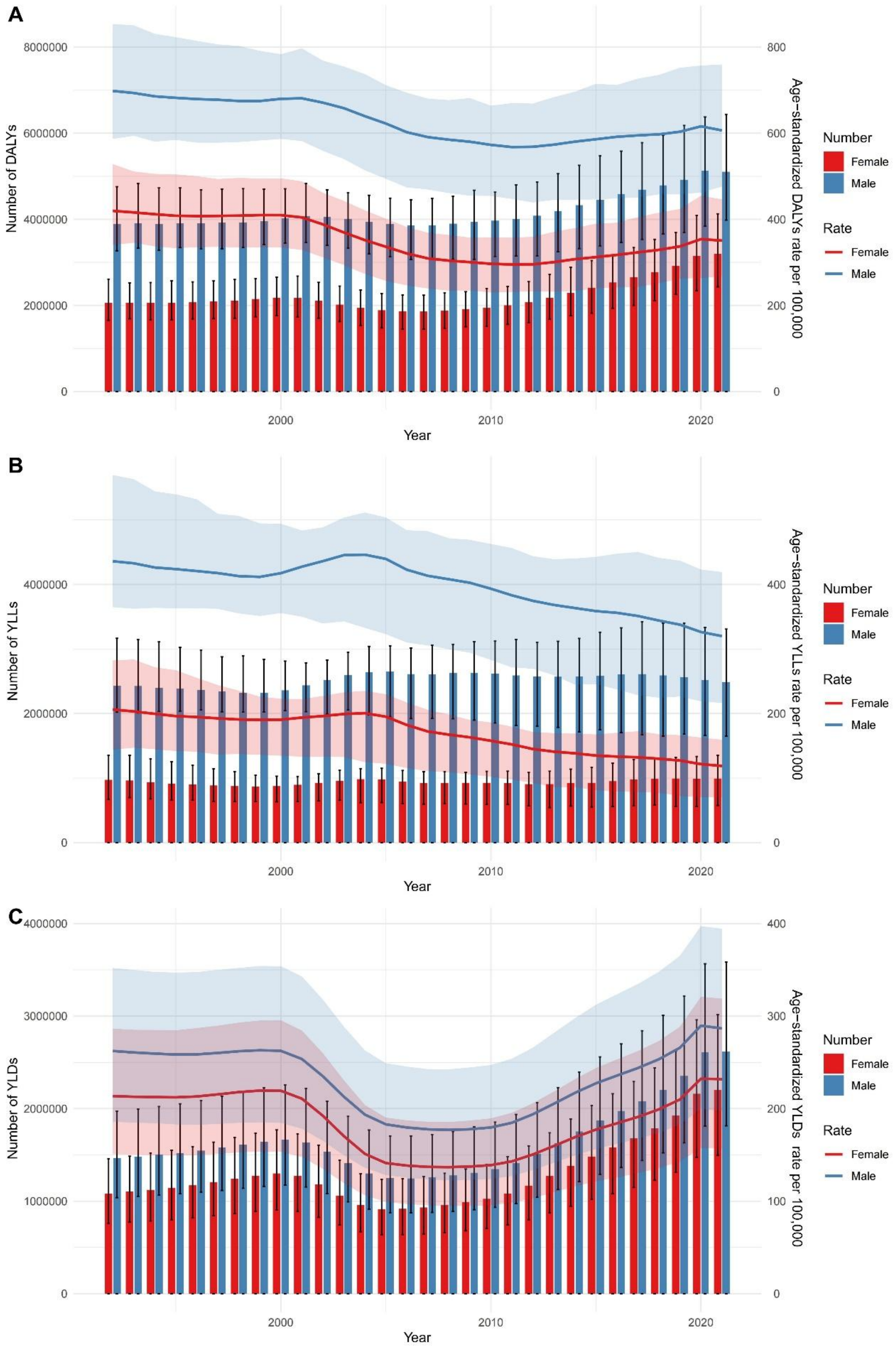

Supplementary Figure 1. Trajectories of the age-standardized rates and all-age numbers of DALYs, YLLs, and YLDs due to falls by sex from 1992 to 2021. (A) DALYs. (B) YLLs. (C)

YLDs.

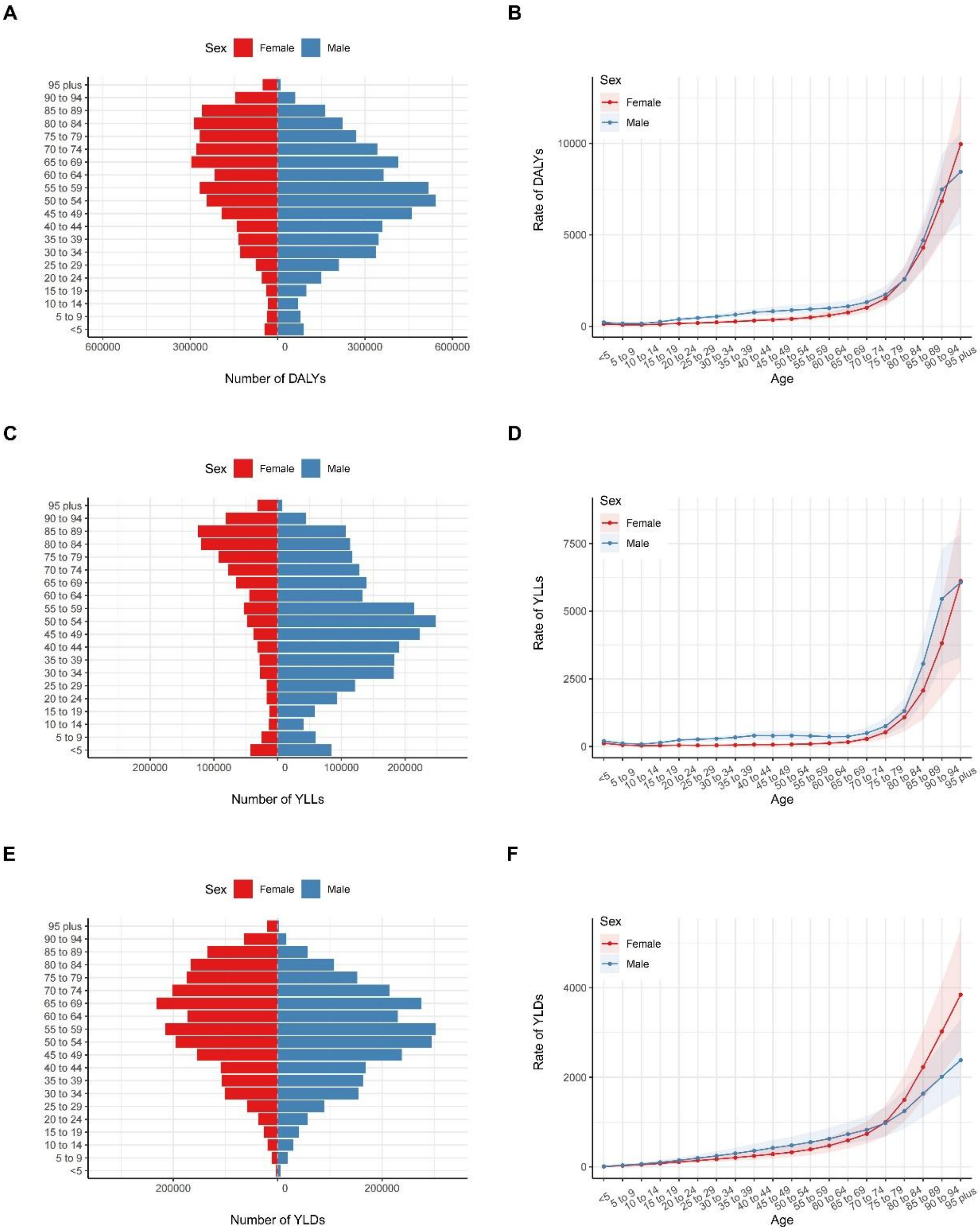

Supplementary Figure 2. Numbers and rates of DALYs, YLLs, and YLDs due to falls among different age groups in China in 2021. (A) number of DALYs. (B) rate of DALYs. (C) number of YLLs. (D) rate of YLLs. (E) number of YLDs. (F) rate of YLDs.

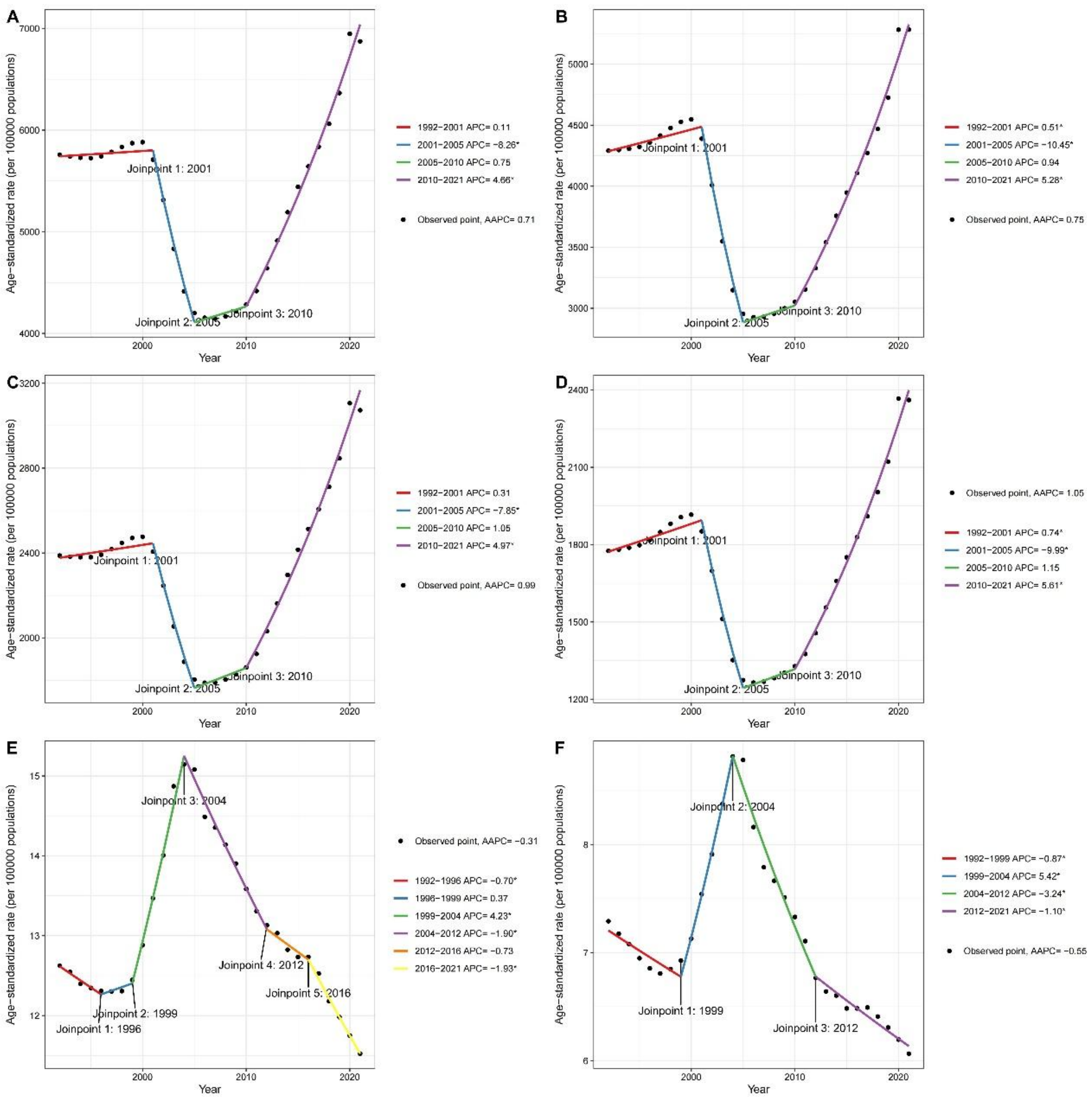

Supplementary Figure 3. Joinpoint regression analysis of ASRs for falls in China over the period 1992 to 2021. (A) prevalence for male, (B) prevalence for female, (C) incidence for male, (D) incidence for female, (E) mortality for male, and (F) mortality for female.

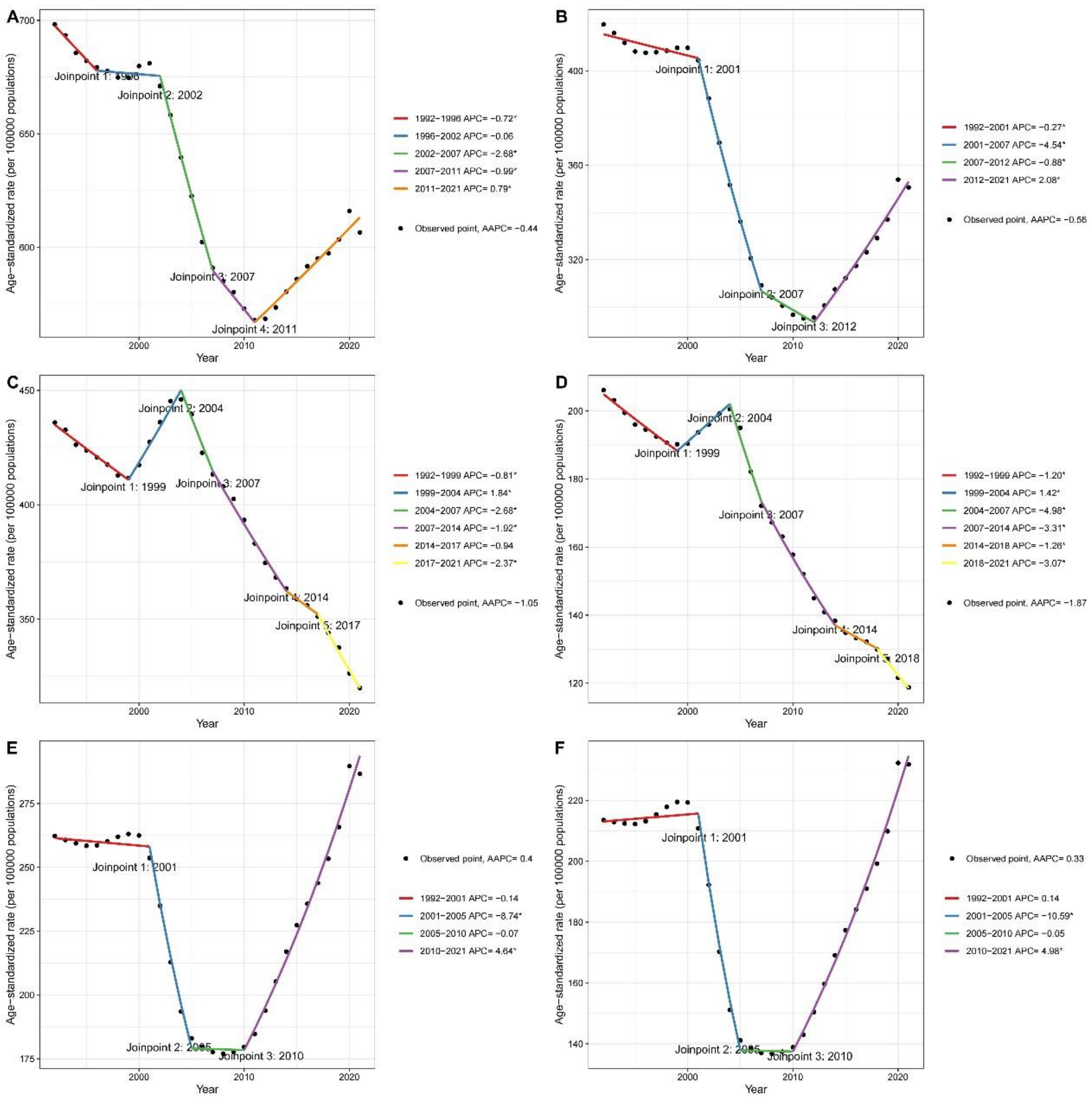

Supplementary Figure 4. Joinpoint regression analysis of ASRs for falls in China over the period 1992 to 2021. (A) DALYs for male, (B) DALYs for female, (C) YLLs for male, (D) YLLs for female, (E) YLDs for male and (F) YLDs for female.
