# Supplementary material for: Burden of falls in China, 1992–2021 and projections to 2030: a systematic analysis for the global burden of disease study 2021
Source: Front Public Health. 2025 Mar 21;13:1538406. doi: 10.3389/fpubh.2025.1538406 (PMC11968356; doi:10.3389/fpubh.2025.1538406)
Supplement: Supplementary file 2 [file Presentation_1.pdf]

Concept Definition

Disability-adjusted life years (DALYs). The sum of years lost due to premature death (YLLs) and years lived with disability (YLDs). One DALY equals one lost year of healthy life.

Years lived with disability (YLDs). Years lived with any short-term or long-term health loss. It is measured by taking the prevalence of the condition multiplied by the disability weight for that condition. Disability weights reflect the severity of different conditions and are developed through surveys of the general public.

Years of life lost (YLLs). Years of life lost due to premature mortality. YLLs are calculated by subtracting the age at death from the longest possible life expectancy for a person at that age. For example, if the longest life expectancy for men in a given country is 75, but a man dies of cancer at 65, this would be 10 years of life lost due to cancer.

Falls includes death or disability resulting from a sudden movement downward due to slipping, tripping, or other unintentional movement that results in a person coming to rest at a lower level or against an object. Included are ICD-9: E880–E886.99, E888E888.9, E929.3; ICD-10: W00–W19.9.

Retrieval strategy

Cause of death or injury

The retrieval strategy for the GBD 2021 is as follows: “GBD estimate”: cause of death or injury; “Measure”: Deaths, DALYs (Disability-Adjusted Life Years), YLDs (Years Lived with Disability), YLLs (Years of Life Lost), Prevalence, Incidence; “Metric”: number, percentage, rate; “Cause”: Falls; “Location”: China; “Age”: all ages, age-standardized, <5 years to >95 years; “Sex”: both, female, male; “Year”: from 1992 to 2021.

Cause of death or injury

The retrieval strategy for the GBD 2021 is as follows: “GBD estimate”: cause of death or injury; “Measure”: Deaths, DALYs (Disability-Adjusted Life Years), YLDs (Years Lived with Disability), YLLs (Years of Life Lost), Prevalence, Incidence; “Metric”: rate; “Cause”: Falls; “Location”: China, United States of America, India, Global; “Age”: age-standardized; “Sex”: both, female, male; “Year”: from 1992 to 2021.

Risk factor

The retrieval strategy for the GBD 2021 is as follows: “GBD estimate”: Risk factor; “Measure”: DALYs (Disability-Adjusted Life Years); “Metric”: percentage; “Risk”: Smoking, Occupational injuries, Low bone mineral density, Alcohol use; “Cause”: Falls; “Location”:

China; “Age”: age-standardized; “Sex”: both, female, male; “Year”: from 1992 to 2021.

Injuries by nature

The retrieval strategy for the GBD 2021 is as follows: “GBD estimate”: Injuries by nature; “Measure”: YLDs (Years Lived with Disability); “Metric”: rate; “Risk”: Amputation of lower limb, unilateral, Fracture of femur, other than femoral neck, Fracture of hip, Fracture of patella, tibia or fibula, or ankle, Fracture of pelvis, Fracture of vertebral column, Minor TBI, Moderate/Severe TBI, Spinal cord lesion at neck level, Spinal cord lesion below neck level; “Cause”: Falls; “Location”: China; “Age”: <5 years to >95 years; “Sex”: both; “Year”: 2021.

In the GBD database, we selected the China field, which includes the Hong Kong Special Administrative Region and the Macao Special Administrative Region, but did not select Taiwan (Province of China).
